# Characteristics of breast cancer patients tested for germline *BRCA1/2* mutations by next‐generation sequencing in Ramathibodi Hospital, Mahidol University

**DOI:** 10.1002/cnr2.1664

**Published:** 2022-07-01

**Authors:** Songporn Oranratnachai, Watchalawalee Yamkaew, Atchara Tunteeratum, Thongchai Sukarayothin, Nareenart Iemwimangsa, Ravat Panvichien

**Affiliations:** ^1^ Division of Medical Oncology, Department of Medicine, Faculty of Medicine Ramathibodi Hospital Mahidol University Bangkok Thailand; ^2^ Division of Medical Genetics, Department of Medicine, Faculty of Medicine Ramathibodi Hospital Mahidol University Bangkok Thailand; ^3^ Breast and Endocrine Surgery Unit, Department of Surgery, Faculty of Medicine Ramathibodi Hospital Mahidol University Bangkok Thailand; ^4^ Center for Medical Genomics Ramathibodi Hospital Mahidol University Bangkok Thailand

**Keywords:** *BRCA1/2* mutations, breast cancer, luminal subtype, next‐generation sequencing (NGS), prognosis

## Abstract

**Introduction:**

Germline mutations in *BRCA1/2* are the most common cause of hereditary breast and ovarian cancer (HBOC) syndrome. Few studies published during the past decade reported the prevalence of germline *BRCA* mutations in Asian patients with breast cancer. We aimed to assess the prevalence and characteristics of Thai patients with breast cancer with germline *BRCA1/2* mutations.

**Methods:**

We retrospectively reviewed all breast cancer patients who were tested for germline *BRCA1/2* mutations during 2014–2018. *BRCA* mutations were detected using next‐generation sequencing and confirmed using Sanger sequencing. We analyzed the characteristics of patients with or without *BRCA* mutations. Disease‐free survival (DFS) and the associated factors were determined.

**Results:**

Among 67 patients, 12 (18%) were *BRCA1/2* carriers (6 each), 4 (6%) harbored variants of uncertain significance, and 51 (76%) were non‐carriers. We discovered two novel *BRCA2* frameshift mutations (c.2380delA and c.8855dupT). Mean ages at breast cancer diagnosis of *BRCA1*, *BRCA2*, and non‐carriers were 39.8, 46.2, and 42.0 years, respectively. The 12 tumors of *BRCA* carriers were mainly the luminal‐B subtype. Two of these tumors were HER2‐positive luminal‐B, and the triple‐negative subtype was not detected. After adjusting for stages and luminal subtypes, *BRCA* carriers experienced worse 3‐year DFS than non‐carriers (81.5% vs. 90.3%, HR 2.04 [0.64–6.49], *p* = .229). The stage at diagnosis was the sole factor significantly associated with 3‐year DFS (100%, 84.8%, and 72.7%; stages I, II, and III, respectively).

**Conclusion:**

Thai patients with breast cancer with *BRCA1/2* mutations were mainly the luminal‐B subtypes with worse prognosis than those without mutations.

## 1 INTRODUCTION

Breast cancer is the most frequently diagnosed cancer and the leading cause of cancer death among females in most countries.^1^ In Thailand, breast cancer is the third most frequent cancer and the third most common cause of cancer death of both sexes.^2^ Breast cancer, which primarily occurs in women and 1% of men, is typically acquired through multistep accumulations of somatic mutations, whereas 5%–10% of breast cancers are inherited through germline mutations. Germline mutations in the breast cancer susceptibility genes, *BRCA1* and *BRCA2*, are the most common causes of hereditary breast and ovarian cancer (HBOC) syndrome.^3^ A large prospective study found that women who inherit deleterious germline mutations of *BRCA1* or *BRCA2* have very high cumulative risks for developing breast cancer (e.g., risks to 80‐year‐olds are 72% and 69% for *BRCA1* and *BRCA2* carriers, respectively).^4^

The main characteristics of patients with breast cancer with *BRCA* mutations include a family history of breast cancer, younger age at diagnosis, male breast cancer, or multiple tumors (bilateral breast cancer or breast and ovarian cancer) in the same patient.^5^ Furthermore, patients with *BRCA1* mutations are most commonly younger at diagnosis and associated with the triple‐negative breast cancer (TNBC) subtype. In contrast, patients with *BRCA2* mutations are mainly associated with estrogen receptor (ER)‐positive breast cancer.^6^, ^7^, ^8^ Furthermore, a more advanced stage at diagnosis and the presence of multiple foci of breast cancers are more common in patients with *BRCA1* and *BRCA2* mutations than in patients with sporadic tumors. Abundant data show that patients with breast cancer with *BRCA1/2* mutations (*BRCA* carriers) experience higher mortality rates than non‐carriers.^5^, ^8^

*BRCA1* and *BRCA2* are tumor suppressor genes located at chromosomes 17q21 and 13q12.3, respectively.^9^, ^10^, ^11^, ^12^ BRCA1 and BRCA2 proteins are essential for repairing DNA‐double strand breaks (DSBs) by homologous recombination, cell growth regulation, and control of cell division.^3^, ^13^, ^14^ Genetic alterations of these genes occur in 5% of all breast cancers and 15%–25% of familial breast cancers worldwide.^7^, ^15^ The prevalence and phenotype of *BRCA* mutations vary according to country and race. The prevalence of *BRCA* mutations depends on the risk of breast cancer development; namely, a low prevalence in patients with sporadic breast cancer but higher in selected high‐risk cases such as breast cancer with a strong family history, bilateral breast cancer, and multiple organ cancer. Only a few studies have investigated the prevalence of *BRCA* mutations in Asian patients with breast cancer. Data from Korea shows a *BRCA1/2* prevalence of 8.9% in high‐risk patients without family history and 22.3% in patients with family history.^16^ In China, the prevalence of *BRCA1/2* in high‐risk patients is 9.1%, but only 3.5% in sporadic breast cancer patients.^17^ Similarly, there is limited data on the prevalence and characteristics of *BRCA*‐associated breast cancer in Thailand. Only two studies have investigated *BRCA1/2* mutations in selected Thai patients with breast cancer with and without familial history of HBOC.^18^, ^19^ One group of investigators in Thailand used the Multiplex Ligation dependent Probe Amplification (MLPA) method to screen for *BRCA1/2* large genomic rearrangement in Thai patients with familial breast cancer; they only found *BRCA1* alteration in 1% of high‐risk patients with breast cancer.^19^

Before the approval of a poly(adenosine diphosphate [ADP]‐ribose) polymerase (PARP) inhibitor for patients with breast cancer with *BRCA* mutations,^20^ few testing sites were available in Thailand. This is likely explained by the limited number of testing laboratories, the lack of geneticists offering pre‐and post‐test counseling, and costly out‐of‐pocket payments.

The Center for Medical Genomics (CMG) Ramathibodi Hospital started using NGS for routine *BRCA* mutation testing in 2014. The number of *BRCA* mutation testing services in Thailand recently increased since the approval of PARP inhibitors as the treatment for cancers with *BRCA* mutations. The present study explored the prevalence and characteristics of breast cancers in Thai patients tested for germline *BRCA1/2* mutations.

## 2 PATIENTS AND METHODS

### 2.1 Patients

We first screened all patients tested for germline mutations of *BRCA1* and *BRCA2* at the CMG Ramathibodi Hospital from January 2014 to December 2018. We excluded patients with cancers other than breast cancer or patients with breast cancer who enrolled in other clinical studies; we also excluded those whose data in the electronic medical records (EMRs) of Ramathibodi Hospital were unavailable. The EMRs were retrospectively reviewed. We collected demographic data at diagnosis, including sex, age, Eastern Cooperative Oncology Group (ECOG) performance status, and TNM staging according to AJCC 8th edition.^21^ Besides, we also collected tumor characteristics, including pathological subtype, histological grading, ER, progesterone receptor (PR), human epidermal growth factor receptor (HER2), and Ki‐67 status as described in the pathological reports. There are some variations from time to time in the reporting systems for ER and PR immunohistochemistry (IHC) staining. In the present study, ER and PR were considered negative if the nuclei of tumor cells were stained <1%. HER2 expression was routinely classified as negative (0, 1+), equivocal (2+), or positive (3+) via IHC staining intensity. Furthermore, fluorescence in situ hybridization (FISH) was performed to confirm and document registration for trastuzumab reimbursement. Luminal subtypes were classified according to the Saint Gallen Guidelines 2015.^22^ Treatments and outcomes such as surgical management, adjuvant therapies, recurrent/metastasis of disease, secondary primary cancer, and death were also collected.

The Ethics Committee on Human Rights related to research involving human subjects, Faculty of Medicine, Ramathibodi Hospital, Mahidol University approved this study (MURA2019/988).

### 2.2 Analysis of germline mutations in *BRCA1* and *BRCA2* detected by NGS

Genomic DNA was extracted from the peripheral blood of patients. Analyses of germline mutations of *BRCA1* and *BRCA2* were performed using NGS at the CMG. All coding regions in *BRCA1* and *BRCA2* were sequenced, and aligned with the Homo sapiens genome assembly GRCh37 (hg19) published by the Genome Reference Consortium.^23^, ^24^ Sequence variants were classified in decreasing order of clinical importance as “Pathogenic,” “Likely pathogenic,” “Variants of Uncertain Significance (VUS),” “Likely benign,” or “Benign,” according to the American College of Medical Genetics and Genomics (ACMG) Standards and Guidelines.^25^ Selected variants identified as “Pathogenic” or “Likely pathogenic” were verified using Sanger sequencing. All Sanger sequencing results confirmed that the selected Pathogenic/Likely pathogenic variants were genuinely present in the samples.

### 2.3 Statistical analysis

Descriptive statistical analysis was used to describe patients' characteristics. Clinical characteristics were expressed as numbers and percentages for categorical variables, and continuous data were expressed as the mean ± standard deviation (SD). Comparisons of characteristics between carriers of *BRCA1/2* and non‐carriers were performed using Fisher's exact test for categorical variables and one‐way analysis of variance for continuous variables; *p*‐value <.05 indicates a significant difference. Disease‐free survival (DFS) was defined as the time from the date of diagnosis to the date of the first event (local recurrence or distant metastasis, second primary cancer, or death from any cause). Patients who were alive without disease recurrence were censored at the cut‐off date (December 31, 2019). Median DFS was estimated using the Kaplan–Meier method. To identify prognostic factors of DFS, we applied Cox regression analysis. *BRCA* status, stage at diagnosis, and luminal subtypes, along with variables with *p*‐value less than .1 from the univariate Cox regression model, were adjusted by multivariate analysis. Stata software, version 16, was applied to perform all analyses.^26^

## 3 RESULTS

### 3.1 Pathogenic germline mutations of BRCA1/2

**FIGURE 1:**
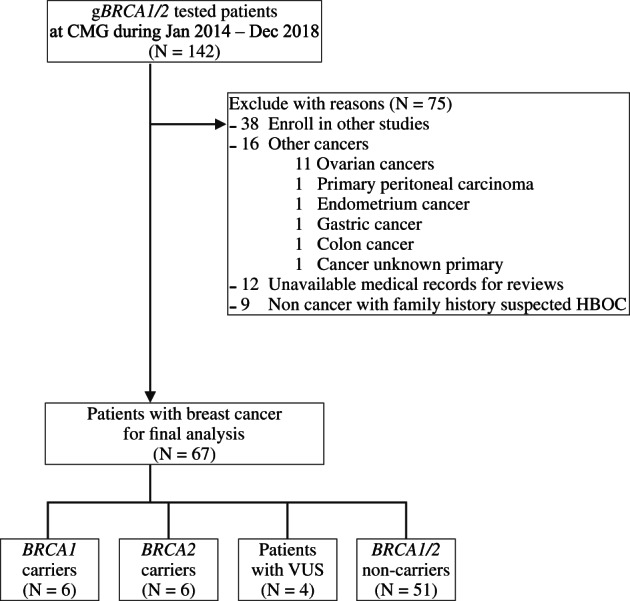
Patient selection

One hundred forty‐two patients had been tested for germline mutations of *BRCA1/2* using NGS at CMG from January 2014 to December 2018. Seventy‐five patients were excluded for reasons, as described in Figure 1. Finally, 67 patients with breast cancer were included in this study. The majority of the included patients (51, 76.1%) were at high risk for HBOC (diagnosed with breast cancer at ≤40 years or TNBC at ≤60 years, male breast cancer, bilateral breast cancer, or multiple organ cancers).

**FIGURE 2:**
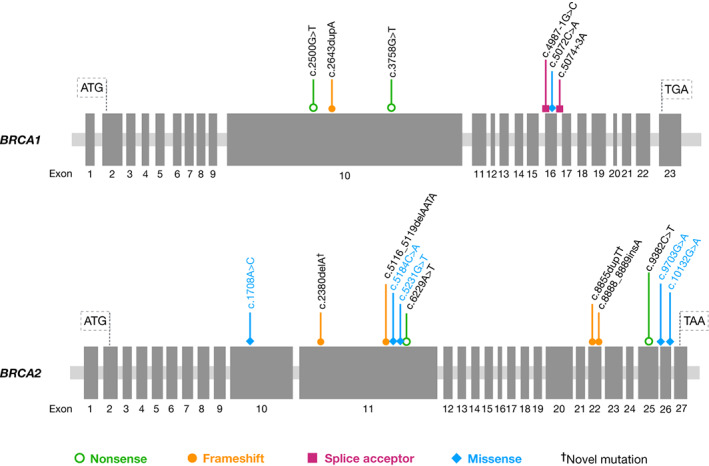
Diagram of *BRCA1* and *BRCA2* genes, indicating the position of pathogenic variants identified here. Exons are indicated by boxes and numbered according to the locus reference genomic (LRG) description. The ATG translation initiation sites and termination codons are indicated by longer lines. Black letters = pathogenic mutation in *BRCA1* and *BRCA2*, Blue letters = VUS in *BRCA2*.

Pathogenic *BRCA* mutations confirmed using Sanger sequencing were detected in 12 (17.9%) patients (6 *BRCA1* carriers and 6 *BRCA2* carriers). VUS were detected in 4 (6.0%) patients, and the others were *BRCA* non‐carriers. The confirmed 12 *BRCA1/2* mutations included five frameshifts, four nonsense, two at splice acceptor positions, and one missense. The positions of these *BRCA1/2* mutations are shown in Figure 2. The mutations of *BRCA2* were more widely distributed across exonic regions than those of *BRCA1*. The six pathogenic mutations of *BRCA1* were located as follows: 3 in exon 10 and 3 in exon 16. The six pathogenic mutations of *BRCA2* were located as follows: 3 in exon 11, 2 in exon 22, and 1 in exon 25.

We discovered two novel frameshift mutations, which have not been reported in any available database (HGMD, ClinVar, dbSNP, gnomAD, etc.), in *BRCA2* [c.2380delA (p.Met794Cysfs) and c.8855dupT (p.Met2952Ilefs)]. We detected another *BRCA2* mutation [c.8888_8889insA (p.Arg2964Lysfs)] reported in previous studies from China and Singapore.^27^, ^28^ The other nine mutations detected here were reported in previous studies of patients residing in Asia, the United States, and Europe (ClinVar^29^ or HGMD databases^30^). The characteristics of the *BRCA* carriers and associated mutations are presented in Table 1. We detected five VUS of *BRCA2* in four of 67 (6.0%) patients. All were missense mutations, and one of the patients carried two VUS. VUS were not detectable in *BRCA1* (Figure 2).

**TABLE 1:** Details of breast cancer in *BRCA* carriers

Age (years)	Sex	Histology	Luminal subtype	Stage	Uni/Bilateral	2nd primary cancer	Recurrence/metastasis	*BRCA1/2* mutations				Status
								*BRCA1/2* gene	Nucleotide change	Protein change	Type	
40	F	IDC	Unknown	IA	Unilateral	Ovarian cancer		*BRCA1*	c.5072C>A	p.Thr1691Lys	Missense	Alive
45	F	ILC	HER2− Luminal B	IA	Unilateral			*BRCA1*	c.2643dupA	p.Cys882Metfs	Frameshift	Alive
45	F	IDC	HER2− Luminal B	IIA	Unilateral	Ovarian cancer		*BRCA1*	c.3748G>T	p.Glu1250Ter	Nonsense	Alive
27	F	IDC	HER2− Luminal B	IIB	Unilateral		Liver metastasis	*BRCA1*	c.4987‐1G>C	?	Splice acceptor	Alive
36	F	IDC	HER2− Luminal B	IIB	Unilateral	Ovarian cancer		*BRCA1*	c.2500G>T	p.Gly834Ter	Nonsense	Alive
46	F	IDC	Unclassified	IIB	Bilateral			*BRCA1*	c.5074+3A>G	?	Splice acceptor	Alive
76	F	IDC	HER2− Luminal B	IIB	Unilateral	PPC		*BRCA2*	c.9382C>T	p.Arg3128Ter	Nonsense	Death
37	F	IDC	HER2− Luminal B	IIB	Unilateral			*BRCA2*	c.5116_5119delAATA	p.Asn1706Leufs	Frameshift	Alive
35	F	IDC	HER2− Luminal B	IIB	Bilateral			*BRCA2*	c.2380delA	p.Met794Cysfs	Frameshift	Alive
36	F	IDC	HER2+ Luminal B	IIIA	Bilateral			*BRCA2*	c.8855dupT	p.Met2952Ilefs	Frameshift	Alive
53	F	IDC	HER2− Luminal B	IIIC	Unilateral			*BRCA2*	c.6229A>T	p.Lys2077Ter	Nonsense	Alive
40	F	IDC	HER2+ Luminal B	IIIC	Unilateral		Liver metastasis	*BRCA2*	c.8888_8889insA	p.Arg2964Lysfs	Frameshift	Alive

Abbreviation: F, female; HER2, human epidermal growth factor receptor; IDC, invasive ductal carcinoma; ILC, invasive lobular carcinoma; PPC, primary peritoneal cancer.

### 3.2 Patients' clinical characteristics and DFS

Among 67 patients, 98.5% were female, except one male with breast cancer who was a *BRCA* non‐carrier. The mean age at diagnosis of breast cancer was 42.0 ± 10.3 years (*BRCA1*, 39.8 ± 7.4 years; *BRCA2*, 46.2 ± 16.0 years). Among 10 (14.9%) patients with bilateral breast cancer, 4 had synchronous lesions, and 6 had metachronous lesions. Most patients presented with early‐stage breast cancer, and 50% had stage II disease. *BRCA* carriers and VUS patients tended to be luminal B subtypes, whereas the most common tumor subtype of non‐carriers was TNBC (27.4%), followed by the HER2‐negative luminal‐B subtype (21.6%). Patients' demographic data are presented in Table 2.

**TABLE 2:** Demographic data of breast cancer patients according to *BRCA* status

Characteristics *N* (%)	Total *N* = 67	*BRCA1/2 N* = 12	VUS *N* = 4	Non‐carriers *N* = 51	*p*‐Value
Sex					1.000
Female	66 (98.5)	12 (100)	4 (100)	50 (98.0)	
Male	1 (1.5)	0	0	1 (2.0)	
Age (mean ± SD, years)	42.0 ± 10.3	43.0 ± 12.4	42.0 ± 8.1	41.7 ± 10.1	0.590
Age at diagnosis					1.000
≤40 years	36 (53.7)	7 (58.3)	2 (50.0)	27 (52.9)	
>40 years	31 (46.3)	5 (41.7)	2 (50.0)	24 (47.1)	
Cancer affected: breast cancer only					0.455
Unilateral	57 (85.0)	9 (75.0)	4 (100)	44 (86.3)	
Metachronous bilateral	6 (9.0)	1 (8.3)	0	5 (9.8)	
Synchronous bilateral	4 (6.0)	2 (16.7)	0	2 (3.9)	
ECOG‐PS					0.239
0	66 (98.5)	11 (91.7)	4 (100)	51 (100)	
1	1 (1.5)	1 (8.3)	0	0	
TNM staging (missing = 2)					0.367
0	3 (4.6)	0	0	3 (6.1)	
I	19 (29.2)	2 (16.7)	0	17 (34.7)	
II	32 (49.2)	7 (58.3)	3 (75.0)	22 (44.9)	
III	11 (16.9)	3 (25.0)	1 (25.0)	7 (14.3)	
ER (missing = 5)					0.072
Negative	22 (35.5)	1 (9.1)	1 (25.0)	20 (42.5)	
Positive	40 (64.5)	10 (90.9)	3 (75.0)	27 (57.5)	
PR (missing = 7)					0.120
Negative	21 (35.0)	1 (10.0)	1 (25.0)	19 (41.3)	
Positive	39 (65.0)	9 (90.0)	3 (75.0)	27 (58.7)	
HER2 (missing = 9)					0.159
Negative	40 (69.0)	8 (80.0)	1 (25.0)	31 (70.4)	
Equivocal	1 (3.4)	0	1 (25.0)	1 (2.3)	
Overexpression	16 (27.6)	2 (20.0)	2 (50.0)	12 (27.3)	
Ki‐67 (missing = 14)					1.000
<20%	7 (13.2)	1 (11.1)	0	6 (15.0)	
≥20%	46 (86.8)	8 (88.9)	4 (100)	34 (85.0)	
Luminal subtypes					0.058
Luminal‐A	6 (9.0)	0	0	6 (11.8)	
HER2– Luminal‐B	19 (28.3)	8 (66.6)	0	11 (21.6)	
HER2+ Luminal‐B	13 (19.4)	2 (16.7)	2 (50.0)	9 (17.6)	
TNBC	15 (22.4)	0	1 (25.0)	14 (27.4)	
HER2+ Non‐luminal	3 (4.5)	0	0	3 (5.9)	
Unclassified	11 (16.4)	2 (16.7)	1 (25.0)	8 (15.7)	
Surgery (missing = 1)					0.257
Mastectomy	51 (77.3)	11 (91.7)	4 (100)	36 (72.0)	
BCS	15 (22.7)	1 (8.3)	0	14 (28.0)	
Adjuvant chemotherapy					0.639
No	20 (29.8)	2 (16.7)	1 (25.0)	17 (33.3)	
Yes	47 (70.2)	10 (83.3)	3 (75.0)	34 (67.7)	
Hormone therapy					0.653
No	24 (35.8)	3 (25.0)	1 (25.0)	20 (39.2)	
Yes	43 (64.2)	9 (75.0)	3 (75.0)	31 (60.8)	
Adjuvant Radiation					0.261
No	29 (43.3)	5 (41.7)	0	24 (47.1)	
Yes	38 (56.7)	7 (58.3)	4 (100)	27 (52.9)	

Abbreviations: BCS, breast conservation surgery; ECOG‐PS, Eastern Cooperative Oncology Group‐Performance status; ER, estrogen receptor; HER2, human epidermal growth factor receptor; PR, progesterone receptor; SD, standard deviation; TNBC, triple‐negative breast cancer.

Surgery was performed on 98.5% of patients, including mastectomy (*n* = 51) and breast‐conserving surgery (BCS; *n* = 15). Almost all *BRCA* carriers underwent mastectomy, except one who underwent BCS and was subsequently found to harbor a *BRCA* mutation. Adjuvant radiotherapy was administered to 56.7% of patients for locally advanced breast cancer (T3–T4) and lymph node metastasis. Adjuvant chemotherapy was provided to 66% of patients; 19 (28.4%) patients received only anthracycline‐based chemotherapy, 16 (23.9%) patients received sequential treatment with anthracycline‐based followed by a taxane, two patients received taxane‐based chemotherapy, and seven patients received other regimens. Platinum‐based adjuvant chemotherapy was not used. Patients with hormone receptor‐positive breast cancer underwent adjuvant endocrine therapy. Trastuzumab was used as a 1‐year adjuvant treatment for 16 confirmed HER2‐positive patients.

Among 12 tumors of *BRCA* carriers, eight were the HER2‐negative luminal‐B, two were the HER2‐positive luminal‐B subtype, and two unclassified (whose breast cancer was diagnosed in 1992 and 2003, and the result of hormonal receptor and HER2 were not available). Unexpectedly, the TNBC subtype was not observed among the *BRCA* carriers. The clinicopathological features of *BRCA1* and *BRCA2* carriers were summarized in Supplementary 1.

The results of the subgroup analysis of *BRCA* non‐carriers are shown in Supplementary 2. Overall, there were no significant differences in clinical and pathological characteristics between early‐onset (≤40 years) and late‐onset (>40 years) patients; however, early‐onset patients received adjuvant chemotherapy at a significantly higher frequency compared with late‐onset patients (*p* = .017). Early‐onset patients tended to have higher grades and stages compared with late‐onset patients.

At the cut‐off date (December 31, 2019), the median follow‐up was 2.7 years (range 0.2–17.2 years), 51 patients were disease‐free, 16 experienced a second primary tumor (*n* = 8), distant metastases (*n* = 7), or local recurrence (*n* = 1). Among the seven patients who developed metastasis, two were *BRCA* carriers, 1 had VUS, and four were *BRCA* non‐carriers; six patients received palliative chemotherapy, and one patient received palliative endocrine therapy. One *BRCA* carrier received platinum‐based chemotherapy followed by a PARP inhibitor, and another *BRCA* carrier received anthracycline and cyclophosphamide.

The 3‐year DFS rate was 87.7% (Table 3). *BRCA1/2* carriers had lower 3‐year DFS rates vs non‐carriers, although the difference was not statistically significant (81.5% vs. 90.3%, hazard ratio (HR) 95% confidence interval (CI), 2.04 (0.64–6.49); *p* = .229). Similarly, patients with VUS had inferior 3‐year DFS compared with non‐carriers (79.0% vs. 90.3%; HR (95% CI), 1.60 (0.20–12.99); *p* = .658). TNM staging was a significant prognostic factor for 3‐year DFS, with higher stage associated with lower 3‐year DFS. Three‐year DFS rates were 100%, 84.8%, and 72.7% for stages I, II, and III, respectively, with HR (95% CI), 2.88 (0.73–11.33); *p* = .131 and 6.82 (1.12–41.33); *p* = .037 for stages II and III compared with stage I, respectively.

**TABLE 3:** Cox regression analysis of prognostic factors associated with 3‐year disease‐free survival

Factors	*N*	3‐year DFS (%)	Univariate analysis		Multivariate analysis	
			HR (95% CI)	*p*‐Value	HR (95% CI)	*p*‐Value
BRCA						
Non‐carrier	50	90.3	1		1	
*BRCA1/2*	12	81.5	2.04 (0.64, 6.49)	0.229	2.50 (0.48, 12.97)	0.275
VUS	4	79.0	1.60 (0.20, 12.99)	0.658	1.25 (0.12, 12.81)	0.852
Age						
≤40 years	36	90.8	1			
>40 years	30	84.4	1.19 (0.42, 3.42)	0.741		
T stage						
Tis	3	100	‐			
T1	22	100	1			
T2	30	80.1	4.32 (1.01, 18.42)	0.048		
T3	10	78.8	4.12 (0.90, 18.91)	0.069		
Lymph node metastasis						
Negative	36	90.4	1			
Positive	29	84.3	1.84 (0.58, 5.79)	0.299		
TNM staging						
0	3	100	‐		‐	
I	19	100	1		1	
II	32	84.8	2.88 (0.73, 11.33)	0.131	6.32 (0.90, 44.48)	0.064
III	11	72.7	6.82 (1.12, 41.33)	0.037	16.30 (1.38, 192.81)	0.027
ER status						
Negative	22	76.8	1			
Positive	40	92.1	0.49 (0.16, 1.46)	0.198		
PR status						
Negative	21	76.7	1			
Positive	39	91.7	0.67 (0.20, 2.22)	0.515		
HER2 status						
Negative	40	82.4	1			
Equivocal	2	100	‐			
Overexpression	16	93.8	0.48 (0.10, 2.31)	0.359		
Ki‐67						
<20%	7	85.7	1			
≥20%	46	84.3	0.97 (0.12, 8.10)	0.981		
Luminal subtype						
Luminal‐A	6	83.3	1		1	
HER2− Luminal‐B	15	93.8	0.46 (0.04, 4.78)	0.513	0.14 (0.01, 2.30)	0.171
HER2+ Luminal‐B	13	92.3	0.33 (0.02, 5.50)	0.441	0.14 (0.01, 3.01)	0.212
TNBC	15	65.3	1.21 (0.13, 11.16)	0.868	1.23 (0.13, 11.78)	0.866
HER2+ Non‐luminal	3	100	0.57 (0.03, 10.52)	0.706	0.72 (0.03, 16.67)	0.836
Unclassified	10	100	0.42 (0.04, 4.74)	0.487	0.65 (0.04, 11.87)	0.775
Cancer affected						
Unilateral	56	89.3	1			
Metachronous bilateral	6	66.7	7.44 (2.26, 24.50)	0.001		
Synchronous bilateral	4	100	1.77 (0.21, 14.79)	0.599		
Adjuvant treatment						
No	19	94.1	1			
Yes	47	85.1	2.44 (0.66, 9.03)	0.181		
Surgery treatment						
BCS	15	92.3	1			
Mastectomy	51	86.5	2.07 (0.46, 9.29)	0.342		
Radiation therapy						
No	28	88.9	1			
Yes	38	86.5	1.03 (0.35, 2.97)	0.961		

Abbreviations: BCS, breast conservation surgery; CI, confidence interval; DFS, disease free survival; ER, estrogen receptor; HER2, human epidermal growth factor receptor; HR, hazard ratio; PR, progesterone receptor; T, tumor size; Tis, carcinoma in situ; TNBC, triple‐negative breast cancer; VUS, Variants of uncertain significance.

After adjusting for the TNM stage and luminal subtypes using multivariable Cox regression analysis, *BRCA1/2* carriers had inferior DFS compared with non‐carriers, although the difference was not statistically significant, HR (95% CI), 2.50 (0.48, 12.97); *p* = .275. Similarly, patients with VUS had inferior DFS compared with non‐carriers, HR (95% CI), 1.25 (0.12–12.81); *p* = .852. After adjustments in multivariate analysis, the TNM stage at diagnosis remained significantly associated with 3‐year DFS. Stages II and III had inferior DFS compared with stage I, HR (95% CI), 6.32 (0.90–44.48); *p* = .064 and 16.30 (1.38–192.81); *p* = .027, respectively (Table 3).

## 4 DISCUSSION

Among 67 patients included in the present study, we detected 6 (8.95%) *BRCA1* carriers, 6 (8.95%) *BRCA2* carriers, 4 (6.0%) patients with VUS, as well as 51 (76.1%) non‐carriers. Within 12 *BRCA* carriers, most had tumors with luminal‐B subtypes (8 HER2‐negative luminal‐B, 2 HER2‐positive luminal‐B, and 2 unclassified due to missing data for hormonal receptor and HER2). *BRCA* carriers had inferior 3‐year DFS than non‐carriers, although the difference was not statistically significant after adjusting for TNM stage and luminal subtype. The TNM stage at diagnosis was the only significant factor associated with 3‐year DFS (higher stage lower 3‐year DFS).

The majority of the previous reports from Thailand focused on *BRCA* in ovarian cancer.^31^, ^32^ In contrast, limited data are available regarding *BRCA* mutations in breast cancer among Thai patients. A few studies conducted in Thailand have included breast cancer in their report; one study investigated in highly selective patients with breast and/or ovarian cancer with strong familial history of HBOC,^18^ and another study screened for *BRCA1/2* large genomic rearrangement with MLPA method in high‐risk patients with familial breast cancer.^19^ Our present retrospective study documents the prevalence and clinical characteristics of Thai patients with breast cancer, including *BRCA* carriers, non‐carriers, and those with VUS as detected by NGS. Although most studies on *BRCA1/2* mutations involved non‐Asian populations,^8^, ^15^, ^33^, ^34^ retrospective studies of high‐risk Asian populations showed the frequencies of *BRCA1* and *BRCA2* mutations between 2.3%–18.6% and 2.3%–11.4%, respectively.^7^ Furthermore, data from Korea demonstrated the prevalence of *BRCA1/2* carriers in high‐risk patients with or without familial history of breast cancer between 8.9% and 22.3%,^16^ while the prevalence of *BRCA1/2* carriers in high‐risk Chinese population is 9.1%.^17^ Here, we showed the frequencies of *BRCA1* and *BRCA2* mutations in high‐risk Thai patients at 8.95% and 8.95%, respectively, consistent with the previous studies of Asian and non‐Asian populations.^7^, ^16^, ^17^

It is well accepted that family history, age at diagnosis, and race are predictive factors for the probability of an individual carrying a germline *BRCA1/2* mutation.^35^, ^36^ Whereas the mean age at diagnosis of breast cancer in *BRCA1/2* carriers and non‐carriers was similar in the present study, the age at diagnosis of breast cancer in *BRCA1* carriers was lower than that of *BRCA2* carriers (39.8 vs. 46.2 years), consistent with previous studies showing the age at diagnosis of breast cancer in *BRCA1* and *BRCA2* carriers ranging between 30 and 45 years.^7^, ^37^ In the subject of bilateral breast cancer, *BRCA1/2* carriers have a higher risk of developing contralateral or second primary breast cancer, with long‐term risks ranging from 60% to 70%.^37^ In the present study, we found 3 out of 12 patients (25%) with *BRCA1/2* mutations had bilateral breast cancer (one with metachronous bilateral breast cancer and two with synchronous bilateral breast cancer). This was similar to a previous study in Korea finding 22.1% of *BRCA1/2* carriers with bilateral breast cancer.^38^ In term of breast cancer subtype, *BRCA1* carriers is typically associated with the TNBC subtype, and hormone receptor‐positive breast cancer is common in *BRCA2* carriers.^34^, ^39^, ^40^ HER2 overexpression has been detected only in 0%–8% of *BRCA*‐associated breast cancer, lower than in sporadic breast cancer.^37^ Consistent with previous studies, all of *BRCA2* carriers in our study were associated with luminal‐B tumors. But we did not find TNBC in *BRCA1* carriers from our study. Therefore, we should consider testing for *BRCA* germline mutations in patients suspected of HBOC presenting with luminal subtype breast cancer; this is relevant because *BRCA* carriers are candidates for specific treatments (platinum‐based chemotherapy or PARP inhibitors) which would increase response rates and prolong survival. Such patients with *BRCA* mutations are candidates for screening for *BRCA*‐associated cancer other than breast cancer to detect the early‐stage disease.

The outcomes of breast cancer in *BRCA* carriers were conflicting. Previous systematic reviews and meta‐analysis found significantly worse overall survival (OS) of *BRCA* carriers than non‐carriers, although the difference in recurrence‐free survival is not statistically significant.^41^, ^42^ Other studies conducted in France and Switzerland found significantly superior 5‐year DFS of *BRCA1* carriers than non‐carriers, but not significant for *BRCA2* carriers.^43^ In contrast, a study conducted in Korea found significantly inferior 10‐year DFS and more contralateral breast cancer in *BRCA1/2* carriers than non‐carriers, but no significant difference in 10‐year OS.^44^ Furthermore, a study of Korean patients found that clinical nodal status is the only significant factor associated with DFS, even after adjusting for clinical nodal stage, *BRCA* status, hormonal receptor status, and Ki‐67. Consistent with these findings, adjusted multivariable analysis in the present study revealed that the only significant factor associated with DFS was the stage at diagnosis, whereas *BRCA1/2* carriers had inferior DFS compared with *BRCA* non‐carriers; however, the difference was not statistically significant. In the future, the treatment outcome of *BRCA*‐associate breast cancer should be better due to the availability of PARP inhibitors that have been approved for use as a treatment for advanced germline *BRCA*‐mutated breast cancer^20^, ^45^ and for adjuvant therapy in selected patients with early stage breast cancer who had germline *BRCA* mutation.^46^

The strength of this study is the use of reliable and comprehensive NGS techniques to analyze *BRCA* sequences. Using NGS, novel genetic alterations could be found and graded. However, our study has some limitations, such as its retrospective analysis of patients tested at a single institution which primarily relied on affordable patients despite the indications for germline *BRCA* testing according to the NCCN guideline. Thus, it may not represent the true prevalence of *BRCA* mutation in Thai patients. Moreover, we could not identify a significant association between *BRCA* status and clinical outcomes because of the study's relatively small sample size. A multicenter prospective study with more subjects is required to identify the prevalence of germline *BRCA* mutations in Thai breast cancer patients.

## 5 CONCLUSION

In summary, this study describes the characteristics, treatment outcomes, and prognostic factors of patients with breast cancer treated at a single university hospital in Thailand. We found that breast cancer in *BRCA* carriers was significantly associated with the luminal‐B subtype. For clinical outcome, the difference of DFS between *BRCA1, BRCA2* carriers, and non‐carriers cannot be demonstrated from this small sample size with a short follow‐up cohort. A longer follow‐up of this study is required to determine the long‐term survival outcomes.

## AUTHOR CONTRIBUTIONS

*Methodology, Formal analysis, Visualization, Writing—initial draft, and contributed to every draft thereafter*, S.O.; *Data curation, Visualization, Writing—initial draft, and contributed to every draft thereafter*, W.Y.; *Supervision and contributed to later drafts of the manuscript*, A.T.; *Supervision and contributed to later drafts of the manuscript*, T.S.; *Resource, Formal analysis, and contributed to later drafts of the manuscript*, N.I.; *Conceptualization, Writing—Review and editing, and contributed to later drafts of the manuscript*, R.P. All authors analyzed the results, read, and approved the manuscript for submission.

## CONFLICT OF INTEREST

The authors declare no conflict of interest.

## ETHICS STATEMENT

The Ethics Committee on Research involving Human Subjects of the Faculty of Medicine, Ramathibodi Hospital, Mahidol University, approved this study. As this was a retrospective study of anonymized patients' data, informed consent was not required.

## Supporting information


**Supplementary 1.** Comparison of breast cancer in *BRCA1* and *BRCA2* carriers.

**Supplementary 2.** Clinicopathologic features of breast cancer in *BRCA* noncarriers.

## Data Availability

All data generated or analyzed during this study are included in this published article.
