# Supplementary material for: Characteristics of breast cancer patients tested for germline BRCA1/2 mutations by next‐generation sequencing in Ramathibodi Hospital, Mahidol University
Source: Cancer Rep (Hoboken). 2022 Jul 1;6(1):e1664. doi: 10.1002/cnr2.1664 (PMC9875646; doi:10.1002/cnr2.1664)
Supplement: Supplementary file 1 — Supplementary 1. Comparison of breast cancer in BRCA1 and BRCA2 carriers. [file CNR2-6-e1664-s001.docx]

**Supplementary 1** Comparison of breast cancer in *BRCA1* and *BRCA2* carriers

| **Characteristics** | ***BRCA1***  N = 6 | ***BRCA2***  N = 6 | ***P*-value** |
| --- | --- | --- | --- |
| **Age** (mean ± SD, years) | 39.8 ± 7.4 | 46.2 ± 16.0 | 0.400 |
| **Age at diagnosis** |  |  | 1.00 |
| ≤ 40 years | 3 (50.0) | 4 (66.7) |  |
| > 40 years | 3 (50.0) | 2 (33.3) |  |
| **ER** (Missing = 1) |  |  | 0.455 |
| Negative | 1 (20.0) | 0 |  |
| Positive | 4 (80.0) | 6 (100) |  |
| **PR** (Missing = 2) |  |  | 0.400 |
| Negative | 1 (25.0) | 0 |  |
| Positive | 3 (75.0) | 6 (100) |  |
| **HER2** (Missing = 2) |  |  | 0.467 |
| Negative | 4 (100) | 4 (66.7) |  |
| Equivocal | 0 | 0 |  |
| Overexpression | 0 | 2 (33.3) |  |
| **Luminal subtypes** |  |  | 0.212 |
| Luminal-A | 0 | 0 |  |
| HER2– Luminal-B | 4 (66.7) | 4 (66.7) |  |
| HER2+ Luminal-B | 0 | 2 (33.3) |  |
| TNBC | 0 | 0 |  |
| HER2+ Non-luminal | 0 | 0 |  |
| Unclassified/Unknown | 2 (33.33) | 0 |  |
| **Stage** |  |  | 0.091 |
| I | 2 (33.3) | 0 |  |
| II | 4 (66.7) | 3 (50.0) |  |
| III | 0 | 3 (50.0) |  |

ER, estrogen receptor; HER2, human epidermal growth factor receptor; PR, progesterone receptor; SD, standard deviation; TNBC, triple-negative breast cancer
