# Supplementary material for: Characteristics of breast cancer patients tested for germline BRCA1/2 mutations by next‐generation sequencing in Ramathibodi Hospital, Mahidol University
Source: Cancer Rep (Hoboken). 2022 Jul 1;6(1):e1664. doi: 10.1002/cnr2.1664 (PMC9875646; doi:10.1002/cnr2.1664)
Supplement: Supplementary file 2 — Supplementary 2. Clinicopathologic features of breast cancer in BRCA noncarriers. [file CNR2-6-e1664-s002.docx]

**Supplementary 2** Clinicopathologic features of breast cancer in *BRCA* noncarriers

| **Characteristics** | **Age** ≤ **40 years**  (N = 27) | **Age > 40 years**  (N = 24) | ***P*-value** |
| --- | --- | --- | --- |
| **Sex** |  |  | 0.471 |
| Female | 27 (100) | 23 (95.8) |  |
| Male | 0 | 1 (4.2) |  |
| **Cancer affected** |  |  | 1.000 |
| Unilateral | 23 (85.2) | 21 (87.5) |  |
| Metachronous bilateral | 3 (11.1) | 2 (8.3) |  |
| Synchronous bilateral | 1 (3.7) | 1 (4.2) |  |
| **ECOG performance status** |  |  |  |
| 0 | 27 (100) | 24 (100) |  |
| **Disease status** |  |  |  |
| Disease free | 23 (85.2) | 19 (79.2) | 0.629 |
| Local recurrence | 1 (3.7) | 0 |  |
| Distant metastasis | 1 (3.7) | 3 (12.5) |  |
| Second primary malignancy | 2 (7.4) | 2 (8.3) |  |
| **Pathological type** |  |  | 0.323 |
| DCIS | 2 (7.4) | 2 (8.3) |  |
| IDC | 21 (77.8) | 22 (91.7) |  |
| IMC | 3 (11.1) | 0 |  |
| ILC | 1 (3.7) | 0 |  |
| **Staging** |  |  | 0.393 |
| 0 | 1 (3.7) | 2 (9.1) |  |
| I | 7 (25.9) | 10 (45.4) |  |
| II | 14 (51.9) | 8 (36.4) |  |
| III | 5 (18.5) | 2 (9.1) |  |
| **Luminal subtypes** |  |  | 0.225 |
| Luminal-A | 3 (11.1) | 3 (12.5) |  |
| HER2– Luminal-B | 9 (33.4) | 2 (8.3) |  |
| HER2+ Luminal-B | 5 (18.5) | 4 (16.7) |  |
| TNBC | 7 (25.9) | 7 (29.2) |  |
| HER2+ Non-Luminal | 1 (3.7) | 2 (8.3) |  |
| Unclassified/Unknown | 2 (7.4) | 6 (25.0) |  |
| **Adjuvant chemotherapy** |  |  | 0.017 |
| No | 5 (18.5) | 12 (50.0) |  |
| Yes | 22 (81.5) | 12 (50.0) |  |
| **Surgery treatment** |  |  | 0.723 |
| BCS | 20 (74.1) | 16 (69.6) |  |
| Mastectomy | 7 (25.9) | 7 (30.4) |  |
| **Radiation treatment** |  |  | 0.128 |
| No | 10 (37.0) | 14 (58.3) |  |
| Yes | 17 (63.0) | 10 (41.7) |  |

BCS, breast conservation surgery; DCIS, ductal carcinoma in situ; ECOG, Eastern Cooperative Oncology Group; HER2, human epidermal growth factor receptor; IDC, invasive ductal carcinoma; ILC, invasive lobular carcinoma; IMC, invasive mammary carcinoma; TNBC, triple-negative breast cancer
